# FBXO22 promotes osteosarcoma progression via regulation of FOXO1 for ubiquitination and degradation

**DOI:** 10.1111/jcmm.70021

**Published:** 2024-08-17

**Authors:** He Zhang, Yang Bai, Jiatong Li, Ting Chen, Guanning Shang

**Affiliations:** ^1^ Department of Orthopedics Shengjing Hospital of China Medical University Shenyang Liaoning China; ^2^ Department of Nursing Shengjing Hospital of China Medical University Shenyang Liaoning China

**Keywords:** degradation, FBXO22, FoxO1, osteosarcoma, ubiquitination

## Abstract

Accumulating evidence has demonstrated that F‐box protein 22 (FBXO22) participates in tumour development and progression in various types of human malignancies. However, the functions and detailed molecular mechanisms of FBXO22 in osteosarcoma tumorigenesis and progression remain elusive. In this study, we aimed to determine the effects of FBXO22 on the cell proliferation, migration and invasion of osteosarcoma cells using cell counting kit‐8 and Matrigel Transwell approaches. Moreover, we explored the molecular mechanisms by which FBXO22 mediated oncogenesis and progression in osteosarcoma via Western blotting, immunoprecipitation and ubiquitination. We found that FBXO22 depletion inhibited the proliferation, migration and invasion of osteosarcoma cells, whereas FBXO22 overexpression increased the proliferation and motility of osteosarcoma cells. Mechanistically, FBXO22 promoted the ubiquitination and degradation of FoxO1 in osteosarcoma cells. FBXO22 depletion reduced cell proliferation and motility via regulation of FoxO1. Taken together, our findings provide new insight into FBXO22‐induced osteosarcoma tumorigenesis. The inhibition of FBXO22 could be a promising strategy for the treatment of osteosarcoma.

## 1 INTRODUCTION

Osteosarcoma is a common primary bone tumour that often develops in children and adolescents as well as in older adults (more than 60 years of age).^1^ In general, 10%−15% of osteosarcoma patients present metastasis at the time of diagnosis. Localized osteosarcoma often has good treatment outcomes; however, osteosarcoma patients with metastasis have poor survival rates.^2^, ^3^, ^4^ In advanced osteosarcoma, neoadjuvant chemotherapy and resection result in improved patient survival.^5^ Nevertheless, acquired drug resistance hinders chemotherapeutic efficacy in osteosarcoma.^6^, ^7^ Recently, immunotherapeutic drugs, which are not highly effective, have been used for the treatment of osteosarcoma.^8^, ^9^, ^10^ Thus, it is necessary to identify novel targets for improving targeted therapy for osteosarcoma.

Ubiquitination is a type of posttranslational modifications that can be orchestrated by three ligases, including ubiquitin‐activating enzymes (E1s), ubiquitin‐conjugating enzymes (E2s) and ubiquitin ligases (E3s).^11^, ^12^ SKP1‐cullin1‐F‐box protein E3s constitute one of the cullin‐RING‐E3 ligase families.^13^ F‐box proteins are known to participate in carcinogenesis by targeting their specific substrates.^14^, ^15^ Accumulating evidence has indicated that F‐box protein 22 (FBXO22) participates in tumour development and progression in various types of human malignancies.^16^, ^17^ High FBXO22 expression is correlated with poor overall survival in numerous cancers.^18^ One study revealed that FBXO22 facilitates hepatocellular carcinoma (HCC) progression by regulating Kruppel‐like transcription factor 4 for destabilization.^19^ Another study reported that FBXO22 targets p21 for degradation and enhances HCC development.^20^ In addition, depletion of FBXO22 retarded cell invasion, migration and angiogenesis via modulation of the hypoxia inducible factor 1 subunit alpha and vascular endothelial growth factor (VEGF) pathways in melanoma.^21^ In lung cancer cells, FBXO22 overexpression inactivates liver kinase B1 and enhances lung cancer cell growth.^22^ Similarly, nuclear factor erythroid‐related factor 2 accelerated tumour metastasis via the suppression of FBXO22‐mediated degradation of BTB domain and CNC homologue 1 in lung cancer.^23^

In osteosarcoma cells, Hou and co‐workers reported that the lncRNA small nucleolar RNA host gene 14 (SNHG14) accelerated tumour progression by targeting miR‐433‐3p and FBXO22 in osteosarcoma.^24^ Knockdown of FBXO22 inhibited the proliferation and motility of 143B and SaOS‐2 osteosarcoma cells.^24^ Nevertheless, the function and detailed molecular mechanisms of FBXO22 are elusive in osteosarcoma, and further studies are needed to determine whether FBXO22 might be a potent target for therapy in osteosarcoma patients. Therefore, in this study, we aimed to determine the effects of FBXO22 on the proliferation, migration and invasion of osteosarcoma cells. Moreover, we explored the molecular mechanisms by which FBXO22 mediated oncogenesis and progression in osteosarcoma.

## 2 MATERIALS AND METHODS

### 2.1 Cell culture

The U2OS and MG63 osteosarcoma cell lines were maintained in Dulbecco's modified Eagle's medium supplemented with 10% fetal bovine serum (FBS) and 100 U/mL penicillin, and 100 μg/mL streptomycin for cell culture. SaOS‐2 cells were maintained in McCoy's 5A modified medium supplemented with 10% FBS, and 100 U/mL penicillin and 100 μg/mL streptomycin. The cells were cultured in 5% CO_2_ at 37°C. All human cell lines had been authenticated using STR (or SNP) profiling within the last 3 years. All experiments were performed with mycoplasma‐free cells.

### 2.2 Quantitative real‐time reverse transcription–PCR

The transfected cells were harvested, and TRIzol reagent was used to extract total RNA. The cDNA reverse transcription kit was subsequently used to reverse transcribe the mRNA to cDNA according to the manufacturer's manual. Polymerase chain reaction (PCR) was carried out using the SYBR Green PCR Master Mix Kit as described previously.^25^

### 2.3 Western blotting assay

The treated osteosarcoma cells were lysed with lysis buffer. The bicinchoninic acid (BCA) method was subsequently used to determine the protein concentration. Protein expression was analysed by sodium dodecyl sulfate‐polyacrylamide gel electrophoresis, and an immunostaining assay was performed according to previous publications.^26^ The following antibodies were used: mouse anti‐FBXO22 (1:1000, sc‐100,736, Santa Cruz), mouse anti‐Ub (1:1000, sc‐166,355, Santa Cruz) and anti‐tubulin (1:5000, T9028, Sigma–Aldrich). ImageJ software was used to obtain the quantitative results.^27^

### 2.4 Transfection assays

The osteosarcoma cells were cultured in 6‐well plates overnight. The cells were then transfected with various plasmids using Lipofectamine 2000. The FBXO22 short hairpin RNA (shRNA), FBXO22 cDNA plasmid and control vectors were purchased from GenePharma Company (Shanghai, China). Cell viability, invasion and migration were assessed after transfection for different durations as described previously.^28^

### 2.5 CCK‐8 assay

Cell viability was measured using the cell counting kit‐8 (CCK‐8) approach as described previously.^29^ The transfected osteosarcoma cells were cultured in 96‐well plates for different durations. The cells were subsequently maintained at 37°C for 3–4 h after addition of 10 μL of CCK‐8 agent. A microplate reader was used to measure the OD values at 450 nm.

### 2.6 EdU assay

The 5‐ethynyl‐2‐deoxyuridine (EdU) assay was used to detect cell proliferation because EdU can be incorporated into replicating DNA to affect DNA synthesis. The transfected osteosarcoma cells were seeded into 96‐well plates for different durations. The cells were incubated with EdU for 2 h and fixed with 4% formaldehyde for 30 min. Next, the cells were stained with Hoechst 33342 for 30 min. EdU‐positive cells were photographed by fluorescence microscopy. The percentage of viable cells was calculated based on the numbers of EdU‐stained and Hoechst 33342‐stained cells.^30^

### 2.7 Wound healing assay

A wound healing assay was used to determine directional cell migration. The transfected osteosarcoma cells were cultured in 6‐well plates until the confluence reached more than 90%. A 100 μL pipette tip was then used to establish a wound in the cell monolayer at the centre of the plate. After the cells were washed and the cell debris was removed with PBS, the wound area was captured at different times. ImageJ software was used to measure the wound closure distance.^31^

### 2.8 Transwell migration and invasion assays

The transfected osteosarcoma cells were cultured on the 24‐well inserts on the top layers. The membrane of the top layer contained Matrigel for the invasion assay or no Matrigel for the migration assay. The top layers were supplemented with 200 μL of FBS‐free medium, while the bottom layers were supplemented with 500 μL of medium supplemented with 10% FBS. At different incubation times, the migratory and invasive cells that migrated through the membrane were stained and photographed under a microscope.^32^

### 2.9 Protein half‐life assays

The protein half‐life of the transfected cells was analysed. Briefly, osteosarcoma cells were treated with 100 μg/mL cycloheximide for different durations. The cells were subsequently harvested, and protein expression levels were detected via Western blotting.^33^

### 2.10 Immunoprecipitation assay

The osteosarcoma cells were transfected with different plasmids for 20 hours. Then, 10 μM MG132 was added to the cells, which were incubated for 10 hours. The cells were lysed in Immunoprecipitation (IP) lysis buffer. BCA reagent was used to measure the protein concentration. Then, 10 mg of cell lysate was incubated with the primary antibody‐conjugated beads for 4 hours in a cold room. After the immunocomplexes were washed, Western blotting was performed with the indicated antibodies.^34^

### 2.11 In vivo ubiquitination

The osteosarcoma cells were transfected with different plasmids, including His‐Ub. Next, the cells were treated with 10 μM MG132 for 10 h, followed by washing and lysis in IP lysis buffer. Then, 10 mg of the cell lysate was incubated with the primary FoxO1 antibody for 4 h in a cold room. The cell lysate was subsequently incubated with protein A/G plus agarose overnight. Beads were detected by Western blotting to measure ubiquitination.^35^

### 2.12 Mouse xenograft assay

Six‐week‐old BALB/c0nu/nu mice were used for the xenograft assays. Transfected osteosarcoma cells were collected and injected into the nude mice. A Vernier calliper was used to measure the longest and shortest diameters of the tumours every 4 days. The tumour volumes were calculated using the following formula: *L* × *W* ^2^ × 0.52, where *L* represents the longest diameter and *W* represents the shortest diameter. After 32 days, the mice were sacrificed, and the tumours were photographed and weighed. The animal studies were approved by the Animal Experimentation Ethical Committee of China Medical University.

### 2.13 Statistical analysis

The results are presented as the means ± standard deviations compared with the control group. Statistical analysis of multiple groups was performed via two‐way analysis of variance with Tukey's post hoc test. Differences between two groups were compared using student's *t*‐test. A *p* value <0.05 was considered to indicate statistical significance. GraphPad Prism software was used for all statistical analyses.

## 3 RESULTS

### 3.1 FBXO22 depletion inhibits the proliferation of osteosarcoma cells

**FIGURE 1:**
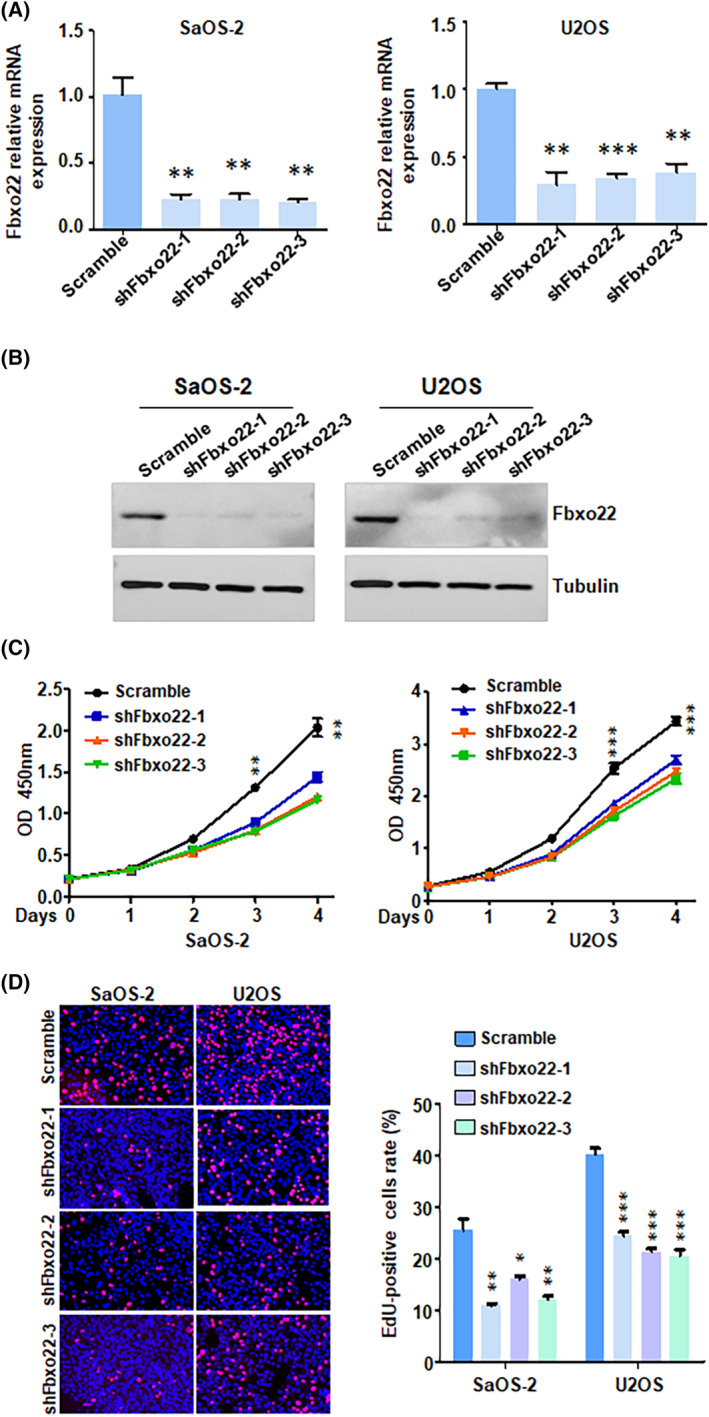
Silencing of F‐box protein 22 (FBXO22) inhibits the proliferation, migration and invasion of osteosarcoma cells. (A) RT‐PCR was performed to measure the FBXO22 mRNA levels in SaOS‐2 and U2OS cells after shFbxo22 transfection for 72 h. (B) Western blotting analysis was performed to measure the protein levels of FBXO22 in SaOS‐2 and U2OS cells after shFbxo22 transfection for 72 h. (C) A CCK‐8 assay was conducted to measure the viability of SaOS‐2 and U2OS cells after shFbxo22 transfection for different durations. (D) An EdU assay was performed to measure the proliferation of SaOS‐2 and U2OS cells after shFbxo22 transfection for 72 h. **p < 0.01, ***p < 0.001, ****p < 0.0001 versus control.

To explore the effect of FBXO22 on the proliferation of osteosarcoma cells, we downregulated the expression of FBXO22 in SaOS‐2 and U2OS cells. The RT–PCR data revealed that FBXO22 mRNA levels were decreased in SaOS‐2 and U2OS cells after FBXO22 shRNA transfection (Figure 1A). Moreover, we performed Western blotting analysis to determine the knockdown efficacy of FBXO22 shRNAs. We found that FBXO22 expression was downregulated in the shFBXO22‐treated groups (Figure 1B). A CCK‐8 assay was conducted to detect the proliferation of osteosarcoma cells after FBXO22 downregulation. The CCK‐8 data revealed that silencing FBXO22 attenuated the proliferation of SaOS‐2 and U2OS cells (Figure 1C). To confirm that cell proliferation was affected by FBXO22 knockdown, an EdU assay was performed in osteosarcoma cells after FBXO22 depletion. Knockdown of FBXO22 reduced the number of EdU‐positive SaOS‐2 and U2OS cells (Figure 1D). Taken together, these findings indicated that depletion of FBXO22 inhibited the proliferation of osteosarcoma cells.

### 3.2 FBXO22 depletion reduces invasion and migration

**FIGURE 2:**
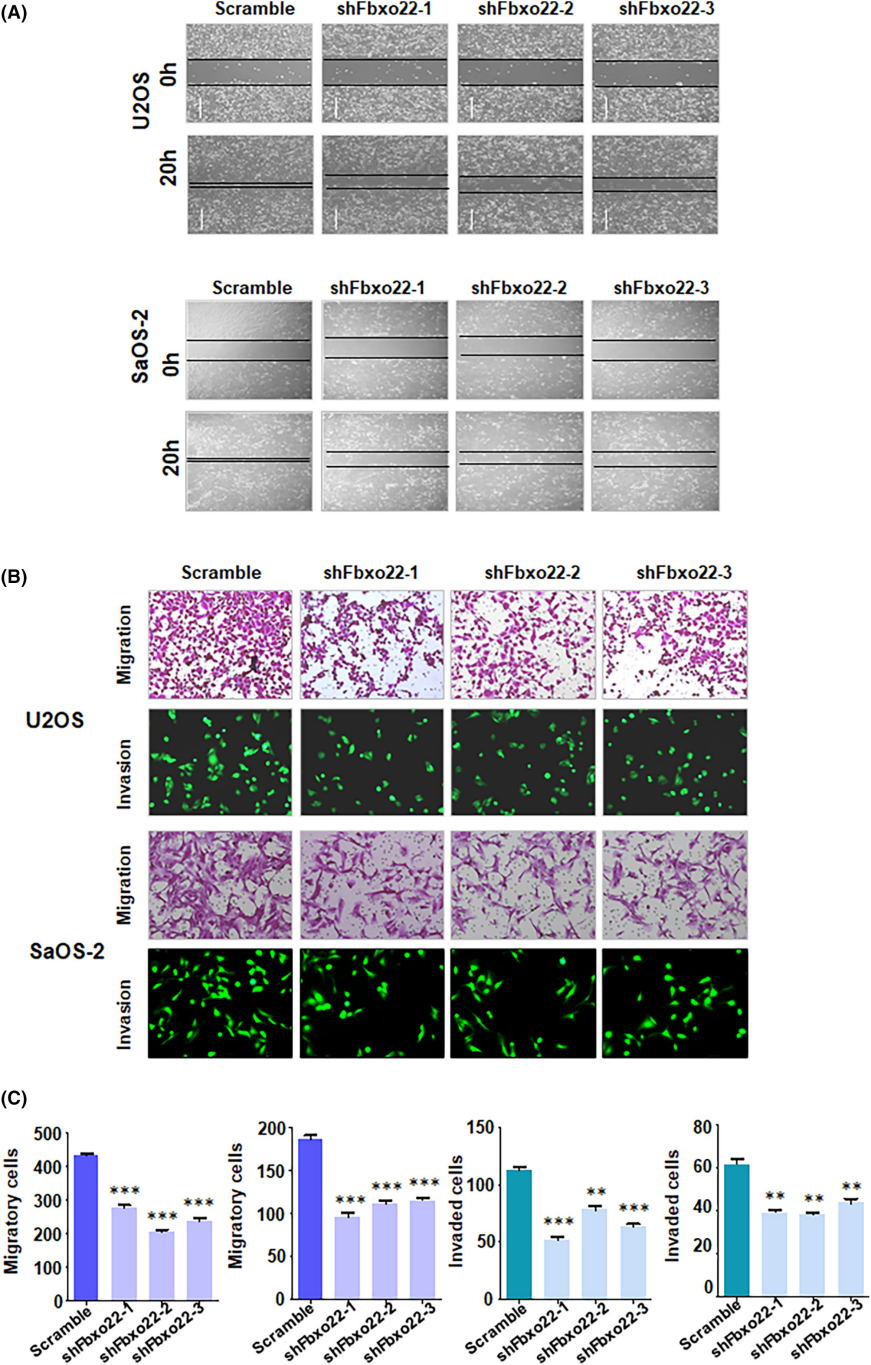
Silencing of F‐box protein 22 (FBXO22) inhibits the migration and invasion of osteosarcoma cells. (A) A wound healing assay was utilized to measure the rate of wound closure in SaOS‐2 and U2OS cells after shFbxo22 transfection for 20 h. (B) Transwell migration and invasion assays were used to detect the migratory and invasive ability of SaOS‐2 and U2OS cells after shFbxo22 transfection for 20 h. (C) Quantitative data are shown in Panel B. **p < 0.01, ***p < 0.001 versus control.

To determine the effect of FBXO22 on the migratory and invasive ability of osteosarcoma cells, we performed wound healing assay and Transwell migration and invasion assays in osteosarcoma cells. Wound healing assays revealed that the closure rate in U2OS and SaOS‐2 cells was attenuated in the shFBXO22‐transfected groups compared with the scramble groups (Figure 2A). Transwell migration assays revealed that, compared with scramble cells, shFBXO22‐transfected cells displayed a reduced migratory ability (Figure 2B,C). Moreover, the Transwell invasion assay results demonstrated a decreased invasive capacity of shFBXO22‐transfected osteosarcoma cells compared with the scramble group (Figure 2B,C). Taken together, these findings indicated that depletion of FBXO22 suppressed the migratory and invasive ability of osteosarcoma cells.

### 3.3 FBXO22 overexpression increases the proliferation and motility of osteosarcoma cells

**FIGURE 3:**
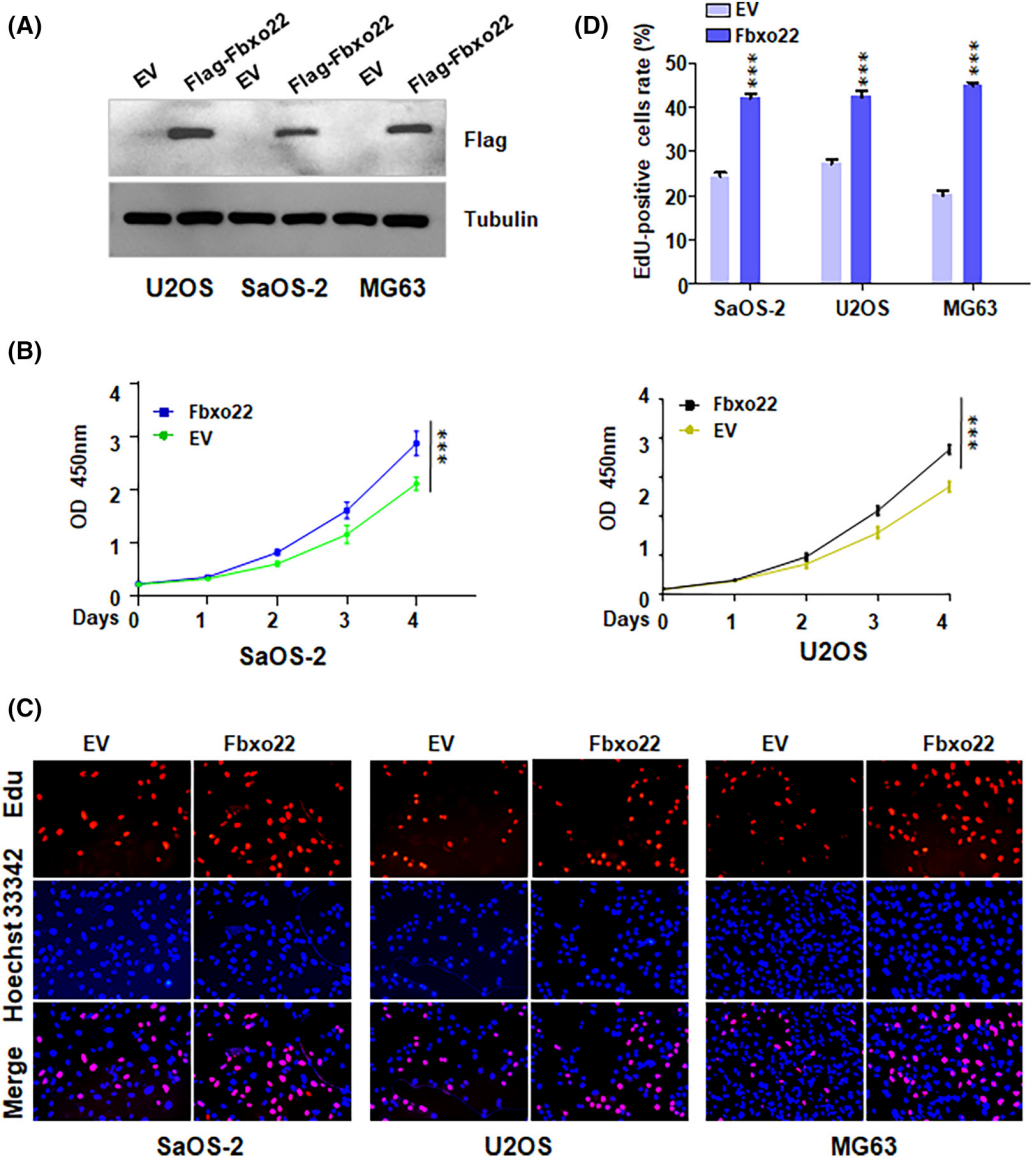
F‐box protein 22 (FBXO22) overexpression promotes the proliferation of osteosarcoma cells. (A) Western blotting analysis was performed to measure the protein levels of FBXO22 in osteosarcoma cells after Fbxo22 cDNA transfection for 72 h. (B) A CCK‐8 assay was conducted to measure the viability of SaOS‐2 and U2OS cells after FBXO22 cDNA transfection for different durations. ***p < 0.001 versus control. (C) An EdU assay was performed to measure the proliferation of osteosarcoma cells after FBXO22 cDNA transfection for 72 h. (D) Right panel: Quantitative data are shown for the EdU assay. ***p < 0.001 versus control.

**FIGURE 4:**
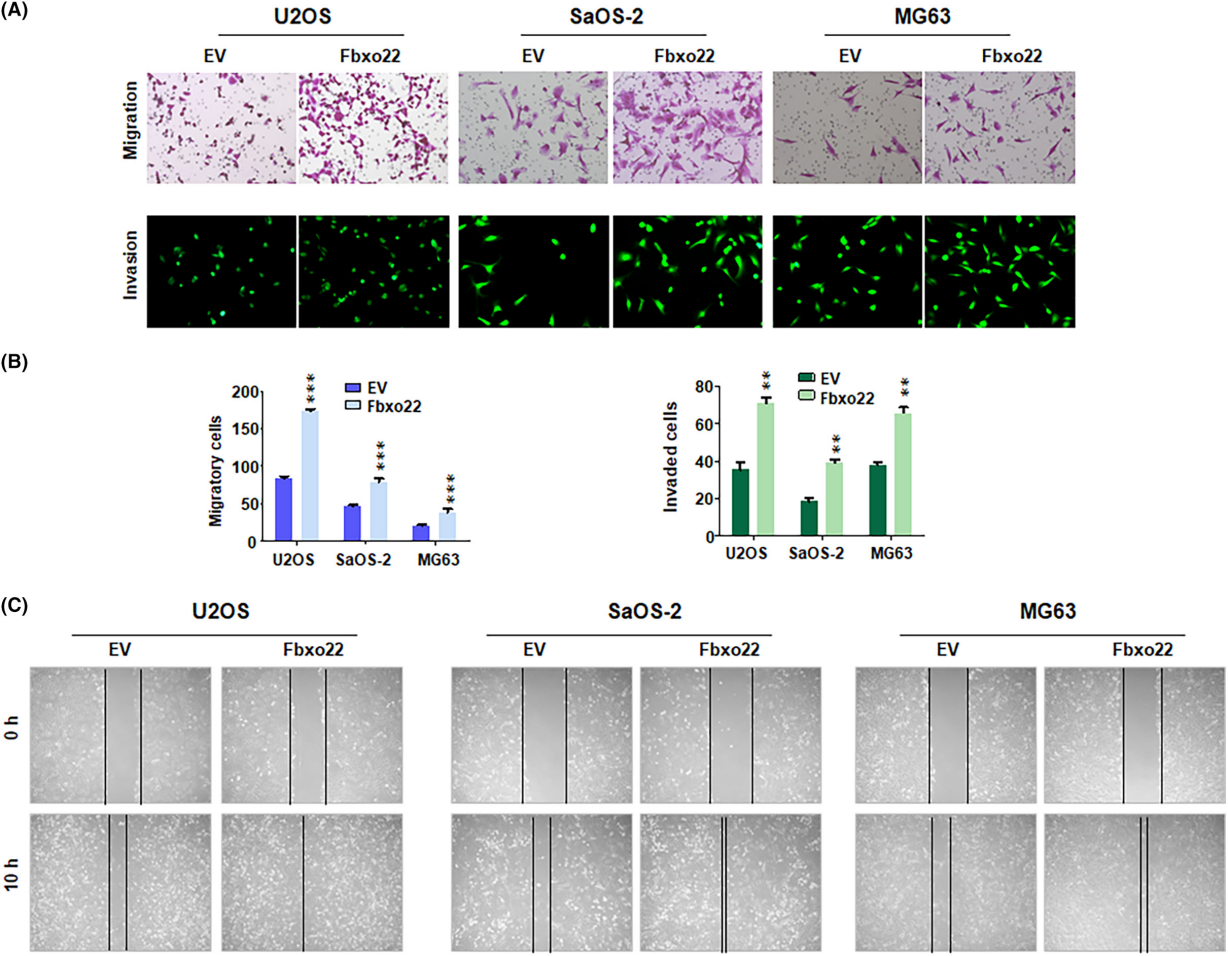
F‐box protein 22 (FBXO22) overexpression promotes the migration and invasion of osteosarcoma cells. (A) Transwell migration and invasion assays were used to detect the migratory and invasive ability of osteosarcoma cells after FBXO22 cDNA transfection for 20 h. (B) Quantitative data are shown for Panel A. **p < 0.01, ***p < 0.001 versus control. (C) A wound healing assay was used to measure the rate of wound closure in osteosarcoma cells after FBXO22 cDNA transfection for 10 h.

Next, we upregulated the expression of FBXO22 in osteosarcoma cells via FBXO22 cDNA transfection. The Western blotting analysis revealed that FBXO22 expression was markedly increased in the FBXO22 cDNA‐transfected U2OS, SaOS‐2 and MG63 cells compared with their empty vector (EV)‐transfected counterparts (Figure 3A). The CCK‐8 assay data revealed that, compared with EV transfection, FBXO22 cDNA transfection increased the proliferation of U2OS and SaOS‐2 cells (Figure 3B). EdU assay data suggested that the overexpression of FBXO22 increased the number of EdU‐positive U2OS, SaOS‐2 and MG63 cells, indicating that the upregulation of FBXO22 promoted the proliferation of osteosarcoma cells (Figure 3C,D). Transwell migration assays revealed that upregulation of FBXO22 enhanced the migratory ability of osteosarcoma cells (Figure 2D). In addition, Transwell invasion assay data confirmed that increased FBXO22 promoted the invasiveness of osteosarcoma cells (Figure 4A,B). Furthermore, the results of the wound healing assay revealed that overexpression of FBXO22 accelerated the rate of wound closure in osteosarcoma cells (Figure 4C). Therefore, FBXO22 overexpression promoted the proliferation, migration and invasion of osteosarcoma cells.

### 3.4 FBXO22 promotes the ubiquitination and degradation of FoxO1

**FIGURE 5:**
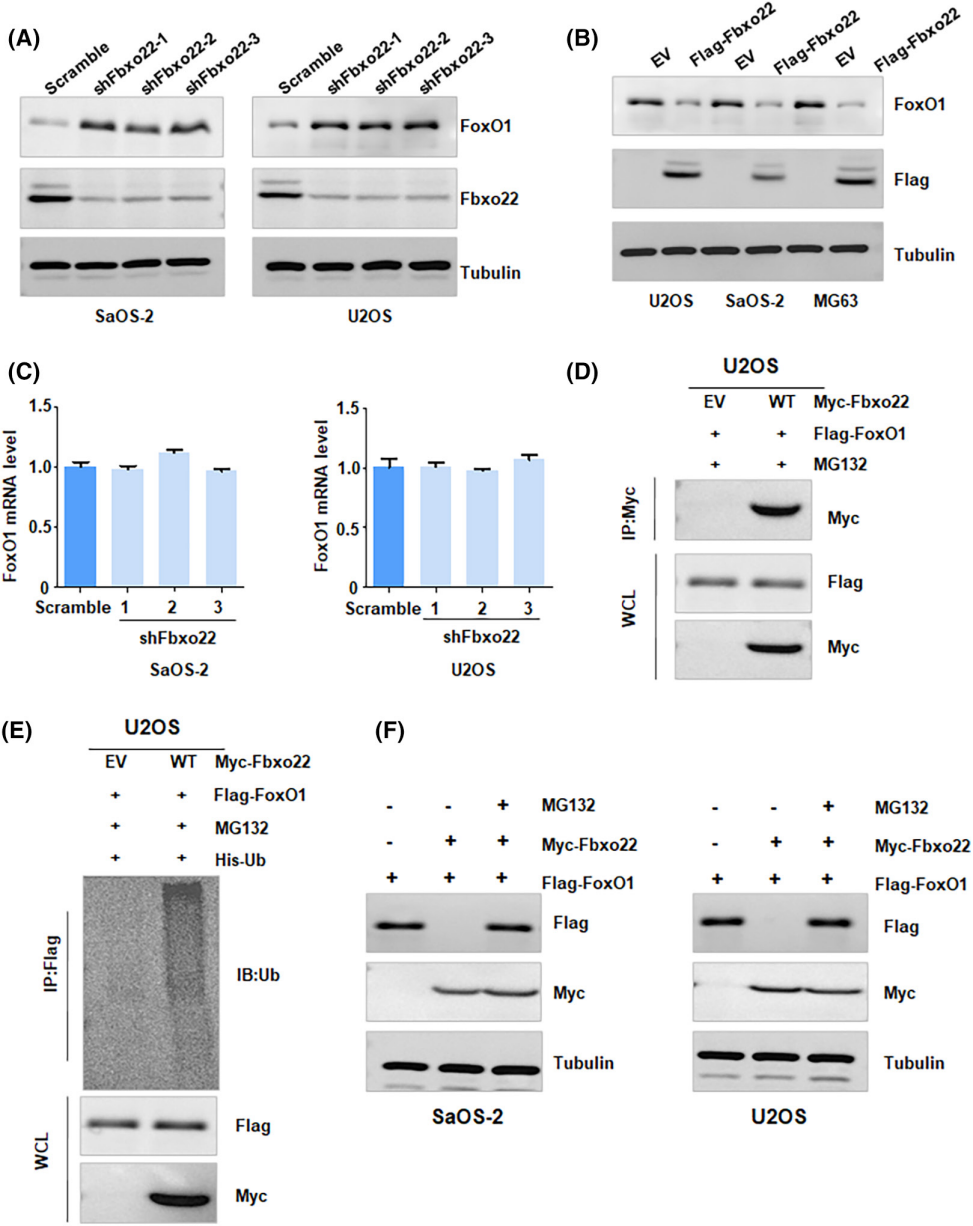
F‐box protein 22 (FBXO22) targets FoxO1 for ubiquitination and degradation. (A) Western blotting analysis was performed to measure the protein levels of FBXO22 and FoxO1 in SaOS‐2 and U2OS cells after shFbxo22 transfection for 72 h. (B) Western blotting analysis was performed to measure the protein levels of FBXO22 and FoxO1 in osteosarcoma cells after Fbxo22 cDNA transfection for 72 h. (C) RT‐PCR was performed to measure the FoxO1 mRNA levels in SaOS‐2 and U2OS cells after shFbxo22 transfection for 72 h. (D) Immunoblotting (IB) of immunoprecipitates and whole‐cell lysates (WCLs) was conducted to detect the interaction between FBXO22 and FoxO1. (E) IB analysis of WCLs and productions of ubiquitination derived from U2OS cells transfected with different constructs. (F) IB analysis of WCLs derived from U2OS and SaOS‐2 cells transfected with different constructs. MG132 (10 μM) was used to treat the cells before harvesting.

**FIGURE 6:**
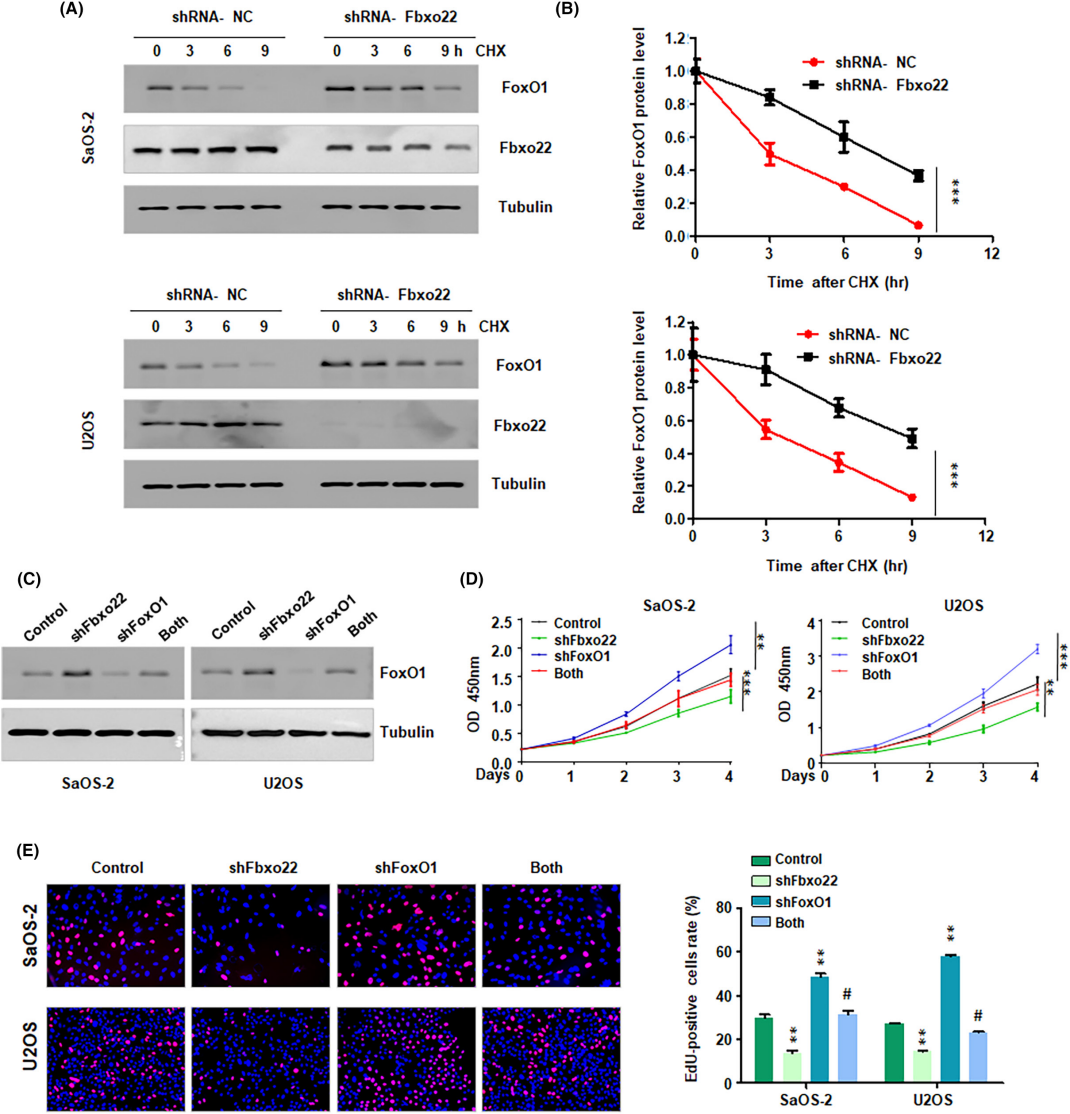
Silencing of F‐box protein 22 (FBXO22) inhibits proliferation by regulating FoxO1. (A) Western blotting analysis was performed to measure the half‐life of FoxO1 protein in SaOS‐2 cells after shFbxo22 transfection for different durations. (B) Quantitative data are shown for the half‐life of FoxO1. (C) Western blotting analysis was performed to measure the protein levels of FoxO1 in SaOS‐2 and U2OS cells after shFbxo22 transfection plus shFoxO1 transfection. (D) A CCK‐8 assay was conducted to measure the viability of SaOS‐2 and U2OS cells after shFbxo22 transfection plus shFoxO1 transfection for different durations. (E) Left panel: An EdU assay was performed to measure the proliferation of SaOS‐2 and U2OS cells after shFbxo22 transfection plus shFoxO1 transfection for 72 h. Right panel: Quantitative data are shown for the EdU assay. **p < 0.01, ***p < 0.001 versus control, # *p* < 0.05 versus shFBXO22 or shFoxO1 alone.

To determine whether FoxO1 is the substrate of FBXO22, we performed a Western blotting analysis in osteosarcoma cells after FBXO22 depletion or overexpression. We observed that depletion of FBXO22 increased the expression of FoxO1 at the protein level in osteosarcoma cells (Figure 5A). Consistently, FBXO22 overexpression reduced FoxO1 protein levels in osteosarcoma cells (Figure 5B). The RT–PCR data indicated that depletion of FBXO22 did not alter the expression of FoxO1 at the mRNA level (Figure 5C). To determine whether FBXO22 interacted with FoxO1, we conducted an IP assay in 293 T and U2OS cells. IP data indicated that FBXO22 could bind to FoxO1 in 293 T and U2OS cells (Figure 5D). Moreover, in vivo ubiquitination data clearly revealed that overexpression of FBXO22 enhanced the ubiquitination of FoxO1 in 293 T and U2OS cells (Figure 5E). Furthermore, MG132 treatment blocked FoxO1 accumulation in osteosarcoma cells after cotransfection with Myc‐FBXO22 and Flag‐FoxO1 (Figure 5F). In addition, half‐life assays demonstrated that FBXO22 knockdown prolonged the half‐life of FoxO1 in U2OS and SaOS‐2 cells (Figure 6A,B). These results suggested that FBXO22 governed the protein stability of FoxO1 in a posttranslational manner.

### 3.5 FBXO22 depletion reduces cell proliferation via regulation of FoxO1

To verify whether FBXO22 knockdown inhibited cell proliferation via the regulation of FoxO1 stability, we cotransfected U2OS and SaOS‐2 cells with shFBXO22 or shFoxO1. We observed that shFBXO22 transfection increased the expression of FoxO1 protein, which partly abolished the shFoxO1‐induced inhibition of FoxO1 in osteosarcoma cells (Figure 6C). The results of the CCK‐8 assay revealed that downregulation of FoxO1 increased the proliferation of U2OS and SaOS‐2 cells (Figure 6D). Knockdown of FBXO22 inhibited the proliferation of osteosarcoma cells, which was abrogated by shFoxO1 transfection (Figure 6D). Similar results were obtained via the EdU assay, which suggested that shFBXO22 reduced the proliferation of osteosarcoma cells and that this phenotype was reversed by shFoxO1 transfection (Figure 6E). Taken together, these findings indicated that the downregulation of FBXO22 repressed osteosarcoma cell proliferation by targeting the FoxO1 pathway.

### 3.6 FBXO22 depletion reduces cell motility by targeting FoxO1

**FIGURE 7:**
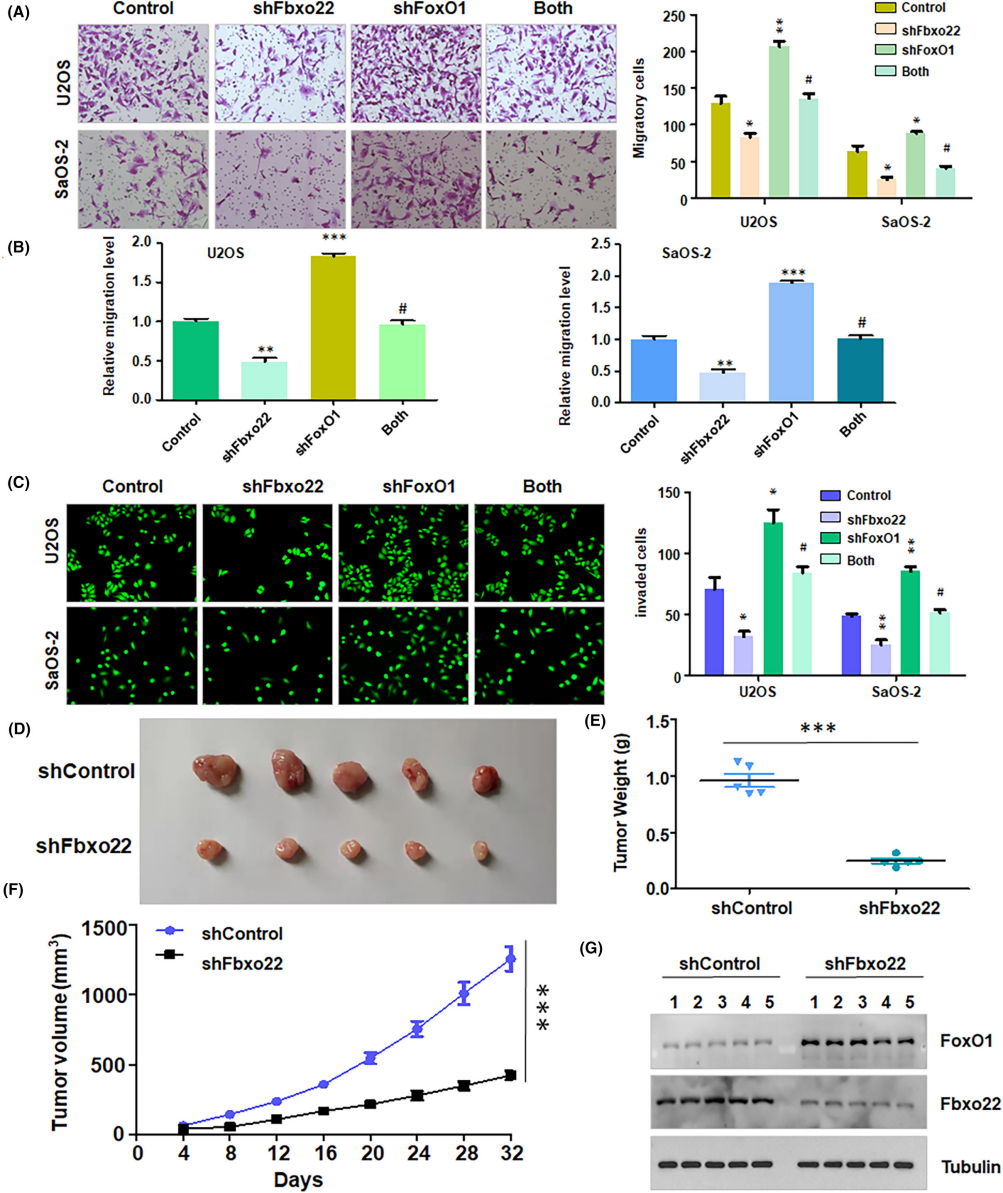
Silencing of F‐box protein 22 (FBXO22) inhibits cell migration, invasion and tumour growth. (A) Left panel: Transwell migration assays were utilized to detect the migratory ability of SaOS‐2 and U2OS cells after shFbxo22 transfection plus shFoxO1 transfection for 20 h. Right panel: Quantitative data are shown for left panel. (B) Quantitative data are shown for the migration ability of osteosarcoma cells. (C) Left panel: Transwell invasion assays were utilized to detect the invasive ability of SaOS‐2 and U2OS cells after shFbxo22 transfection plus shFoxO1 transfection for 20 h. Right panel: Quantitative data are shown for left panel. *p < 0.05, **p < 0.01, ***p < 0.001 versus control, # *p* < 0.05 versus shFBXO22 or shFoxO1. (D) ShFBXO22‐transfected SaOS‐2 cells were injected subcutaneously into BALB/c‐nu/nu mice. After 32 days, the tumours were dissected, and images were obtained. (E) The weights of the dissected tumours are shown. (F) The tumour volumes were measured over the different time periods. (G) Western blotting analysis was performed to measure the expression of FBXO22 and FoxO1 in the dissected tumours. ***p < 0.001 versus control.

To test whether depletion of FBXO22 affected cell motility by regulating FoxO1 in osteosarcoma cells, we performed Transwell migration and invasion assays in U2OS and SaOS‐2 cells after shFBXO22 transfection in combination with shFoxO1 treatment. Transwell migration assays revealed that shFoxO1 treatment enhanced the migratory ability of U2OS and SaOS‐2 cells (Figure 7A). Moreover, the shFBXO22‐induced inhibition of cell migration was abrogated by shFoxO1 treatment in osteosarcoma cells (Figure 7A,B). Furthermore, depletion of FoxO1 accelerated the invasive ability of U2OS and SaOS‐2 cells (Figure 7C). Consistent with these findings, FBXO22 knockdown reduced the invasive ability of osteosarcoma cells, which was abolished by shFoxO1 treatment (Figure 7C). These results suggested that FBXO22 depletion reduced cell motility by targeting FoxO1 in osteosarcoma cells.

### 3.7 FBXO22 knockdown inhibits tumorigenesis in mice

Our in vitro results showed that depletion of FBXO22 reduced the proliferation of osteosarcoma cells. To validate this finding, we used nude mice to determine whether FBXO22 depletion in SaOS‐2 cells retarded tumour growth in vivo. We observed that FBXO22 knockdown inhibited the growth of tumour xenografts in mice (Figure 7D). Tumour weights and volumes were decreased in the FBXO22 depletion group (Figure 7E,F). Moreover, we conducted Western blotting analysis to measure the expression of FoxO1 and FBXO22 in xenograft tumours. We observed that FoxO1 expression was upregulated in FBXO22‐knockdown xenograft tumour tissues (Figure 7G). Therefore, FBXO22 knockdown inhibited tumour growth in mice.

## 4 DISCUSSION

In the present study, we reported that FBXO22 plays a tumour‐promoting role in osteosarcoma cells via regulating the abundance of the FoxO1 protein. Recently, Lin et al. reported that FBXO22 targets p57 for ubiquitination and subsequent degradation and enhances cervical cancer progression.^25^ In cervical cancer cells, ectopic expression of FBXO22 promotes the viability of cervical cancer cells in vitro and facilitates tumour growth in vivo. Ectopic expression of FBXO22 attenuates cell apoptosis and facilitates G1/S phase progression in cervical cancer cells.^25^ In A549 non‐small cell lung cancer (NSCLC) cells, overexpression of FBXO22 increases cell proliferation via the promotion of cyclin‐dependent kinase 4 protein levels and the regulation of phosphatase and tensin homologue (PTEN).^18^ In epithelial ovarian cancer cells, FBXO22 facilitates metastasis and proliferation and represses autophagy by regulating the mitogen‐activated protein kinase (MAPK) and extracellular signal‐regulated kinase (ERK) pathway.^36^ Ge et al. discovered that FBXO22 targets nuclear PTEN for degradation to accelerate oncogenesis.^37^ Consistent with these reports, we demonstrated that FBXO22 could be an oncoprotein involved in osteosarcoma tumorigenesis and progression.

FoxO1, a transcription factor, has been identified as a tumour suppressor in a variety of human cancers.^38^ One study showed that liver X receptor α suppressed the proliferation of osteosarcoma cells via the promotion of FoxO1 expression.^39^ Another study revealed that miR‐135b could accelerate cell proliferation and invasion via the inhibition of FoxO1 in osteosarcoma cells.^40^ Similarly, miR‐374a downregulates the expression of FoxO1 and promotes cell proliferation promotion in human osteosarcoma.^41^ Additionally, miR‐196a enhances cell growth and reduces apoptosis by targeting the PTEN/Akt/FoxO1 pathway in osteosarcoma.^42^ FoxO1 blocks osteosarcoma tumorigenesis through suppression of the Wnt/β‐catenin signalling pathway.^43^ In our study, we found that inhibition of FoxO1 enhanced the proliferation, migration and invasion of osteosarcoma cells. FBXO22 exhibited oncogenic functions in osteosarcoma cells via a reduction in the FoxO1 protein.

Interestingly, several studies have reported that FBXO22 plays an antitumor role in multiple cancer types.^44^, ^45^ For example, FBXO22 reduces tumour metastasis via the suppression of matrix metalloproteinase‐9 (MMP‐9)‐involved invasion and migration and the blockade of VEGF‐induced angiogenesis in renal cell carcinoma.^45^ In fact, FBXO22 does not affect the proliferation of renal cancer cells; however, FBXO22 restricts the invasion and migration of renal cancer cells by abolishing epithelial‐mesenchymal transition (EMT) and increasing the activity of tissue inhibitor of matrix metalloproteinase‐1 (TIMP‐1), leading to the suppression of MMP‐9 expression and activity. Moreover, FBXO22 reduces the secretion of VEGF and impairs tube formation in cells. Li et al. reported that FBXO22 blocks tumour metastasis via modification of lysine demethylase 5A (KDM5A) ubiquitin and modulates H3K4me3 demethylation in triple‐negative breast cancer (TNBC).^44^ FBXO22 attenuates KDM5A expression via ubiquitination, leading to a reduction in H3K4me3 demethylation and upregulation of p16, which inhibits oncogenesis and metastasis in TNBC.^44^ FBXO22 increases the cisplatin sensitivity of tumour cells by mediating the ubiquitination and degradation of CD147 in A549 lung cancer cells.^46^ FBXO22 targets the degradation of the oncoprotein NSD2 in acute lymphoblastic leukaemia cells.^47^ A CRISPR activation screen revealed that FBXO22 degrades endogenous proteins, such as bromodomain‐containing protein 4 (BRD4) and enchinoderm microtubule‐associated protein‐like 4‐anaplastic lymphoma kinase (EML4‐ALK).^48^ Alkylamine‐tethered molecules were found to be required for the recruitment of FBXO22 to degrade FKBP12 protein.^49^ These reports indicate that FBXO22 performs its biological functions in a cancer type‐dependent manner.

Surprisingly, FBXO22 exhibited both antimetastatic functions and protumorigenic effects on breast cancer development and progression.^50^ Another study revealed that FBXO22‐induced degradation of KDM4B influences the activity of selective oestrogen receptor modulators (SERMs) in breast cancer.^51^ Moreover, FBXO22 inhibited tumour cell invasion and metastasis by controlling human homologue of mouse double minute 2 (HDM2) degradation in breast cancer.^52^ These reports suggest a complicated role of FBXO22 in breast cancer progression. Recently, FBXO22 was reported to be critically involved in immunotherapy by targeting programmed death‐ligand 1 (PD‐L1) in tumour cells.^53^ FBXO22 promotes PD‐L1 degradation via an ubiquitination and leads to increased tumour cell sensitivity to DNA damage in NSCLC. Cyclin‐dependent kinase 5 (CDK5) has been shown to increase the expression of PD‐L1 in NSCLC and medulloblastoma.^53^ Silencing of CDK5 increases FBXO22 expression and subsequently reduces PD‐L1 expression in NSCLC. This study revealed that blockade of both CDK5 and PD‐L1 could increase the efficacy of immunotherapy in NSCLC.^53^ One group reported that FoxO1 could interact with and promote the expression of PD‐1 in CD8^+^ T cells during chronic infection.^54^ Since immunotherapies are useful for osteosarcoma treatment,^55^ targeting FBXO22 could be an ideal approach for treating osteosarcoma. However, the relationships among FBXO22, PD‐1, PD‐L1 and FoxO1 in osteosarcoma should be clarified for future immunotherapy application.

FBXO22 was also shown to be regulated by several regulatory factors, such as p53,^56^ the lncRNA SNHG14^24^ and circ_0006282.^57^ Researchers have revealed that noncoding RNAs play essential roles in carcinogenesis, including in osteosarcoma.^58^ He and colleagues have reported that circ_0006282 sponges miR‐155 and elevates FBXO22 expression, contributing to gastric cancer progression.^57^ There is a need to determine the mechanisms responsible for the regulation of FBXO22 in osteosarcoma. Several critical limitations should be mentioned. For example, numerous downstream targets of FBXO22, such as p57, PTEN and HDM2, have been identified. It is unclear whether FBXO22 targets the degradation of p57, PTEN and HDM2 in osteosarcoma, which requires future exploration. However, whether FBXO22 affects the proliferation and motility of osteosarcoma cells by targeting FoxO1 and other proteins, such as p57, PTEN and MMP‐9, is elusive. The associations between FBXO22 and clinical features should be determined in patients with osteosarcoma. Determining the correlation between FBXO22 and FoxO1 in osteosarcoma tissues is critical. Moreover, the development of inhibitors of FBXO22 for the treatment of osteosarcoma is pivotal. In summary, this is the first study to identify FoxO1 as a novel ubiquitin substrate of FBXO22. FBXO22 specifically interacted with FoxO1, and promoted its ubiquitination and subsequent degradation. Overexpression of FBXO22 promoted cell proliferation and migration and invasion. Our findings provide evidence for a novel mechanism by which FBXO22 promotes the degradation of the FoxO1 tumour suppressor in osteosarcoma in part.

## AUTHOR CONTRIBUTIONS

**He Zhang:** Data curation (equal); formal analysis (equal); investigation (equal); methodology (equal); resources (equal); software (equal); writing – original draft (equal). **Yang Bai:** Data curation (equal); formal analysis (equal); investigation (equal); methodology (equal); resources (equal); software (equal); writing – original draft (equal). **Jiatong Li:** Formal analysis (supporting); investigation (supporting); methodology (supporting); resources (supporting); writing – original draft (supporting). **Ting Chen:** Conceptualization (supporting); project administration (supporting); supervision (supporting); validation (supporting); visualization (supporting); writing – review and editing (supporting). **Guanning Shang:** Conceptualization (lead); investigation (equal); project administration (equal); supervision (lead); validation (lead); visualization (lead); writing – review and editing (lead).

## CONFLICT OF INTEREST STATEMENT

The authors have declared that no competing interest exists.

## Data Availability

The datasets are available from the corresponding author on reasonable request.

## References

[1] Meltzer PS , Helman LJ . New horizons in the treatment of osteosarcoma. N Engl J Med. 2021;385(22):2066‐2076. doi:10.1056/NEJMra2103423 34818481

[2] Beird HC , Bielack SS , Flanagan AM , et al. Osteosarcoma. Nat Rev Dis Prim. 2022;8(1):77. doi:10.1038/s41572-022-00409-y 36481668

[3] Moukengue B , Lallier M , Marchandet L , et al. Origin and therapies of osteosarcoma. Cancers (Basel). 2022;14(14):3503. doi:10.3390/cancers14143503 35884563 PMC9322921

[4] Sheng G , Gao Y , Yang Y , Wu H . Osteosarcoma and metastasis. Front Oncol. 2021;11:780264. doi:10.3389/fonc.2021.780264 34956899 PMC8702962

[5] Gill J , Gorlick R . Advancing therapy for osteosarcoma. Nat Rev Clin Oncol. 2021;18(10):609‐624. doi:10.1038/s41571-021-00519-8 34131316

[6] Chen C , Shi Q , Xu J , Ren T , Huang Y , Guo W . Current progress and open challenges for applying tyrosine kinase inhibitors in osteosarcoma. Cell Death Dis. 2022;8(1):488. doi:10.1038/s41420-022-01252-6 PMC9744866 36509754

[7] Garcia‐Ortega DY , Cabrera‐Nieto SA , Caro‐Sanchez HS , Cruz‐Ramos M . An overview of resistance to chemotherapy in osteosarcoma and future perspectives. Cancer Drug Resist. 2022;5(3):762‐793. doi:10.20517/cdr.2022.18 36176756 PMC9511812

[8] Hu Z , Wen S , Huo Z , et al. Current status and prospects of targeted therapy for osteosarcoma. Cells. 2022;11(21):3507. doi:10.3390/cells11213507 36359903 PMC9653755

[9] Zhang Z , Tan X , Jiang Z , Wang H , Yuan H . Immune checkpoint inhibitors in osteosarcoma: a hopeful and challenging future. Front Pharmacol. 2022;13:1031527. doi:10.3389/fphar.2022.1031527 36324681 PMC9618820

[10] Wen Y , Tang F , Tu C , Hornicek F , Duan Z , Min L . Immune checkpoints in osteosarcoma: recent advances and therapeutic potential. Cancer Lett. 2022;547:215887. doi:10.1016/j.canlet.2022.215887 35995141

[11] Senft D , Qi J , Ronai ZA . Ubiquitin ligases in oncogenic transformation and cancer therapy. Nat Rev Cancer. 2018;18(2):69‐88. doi:10.1038/nrc.2017.105 29242641 PMC6054770

[12] Wang W , Liu W , Chen Q , Yuan Y , Wang P . Targeting CSC‐related transcription factors by E3 ubiquitin ligases for cancer therapy. Semin Cancer Biol. 2022;87:84‐97. doi:10.1016/j.semcancer.2022.11.002 36371028

[13] Liu J , Chen T , Li S , Liu W , Wang P , Shang G . Targeting matrix metalloproteinases by E3 ubiquitin ligases as a way to regulate the tumor microenvironment for cancer therapy. Semin Cancer Biol. 2022;86(Pt 2):259‐268. doi:10.1016/j.semcancer.2022.06.004 35724822

[14] Wang Z , Liu P , Inuzuka H , Wei W . Roles of F‐box proteins in cancer. Nat Rev Cancer. 2014;14(4):233‐247. doi:10.1038/nrc3700 24658274 PMC4306233

[15] Xiong HJ , Yu HQ , Zhang J , et al. Elevated FBXL6 activates both wild‐type KRAS and mutant KRAS(G12D) and drives HCC tumorigenesis via the ERK/mTOR/PRELID2/ROS axis in mice. Mil Med Res. 2023;10:68. doi:10.1186/s40779-023-00501-8 38124228 PMC10731709

[16] Cheng J , Lin M , Chu M , Gong L , Bi Y , Zhao Y . Emerging role of FBXO22 in carcinogenesis. Cell Death Dis. 2020;6:66. doi:10.1038/s41420-020-00303-0 PMC7385156 32793396

[17] Johmura Y , Harris AS , Ohta T , Nakanishi M . FBXO22, an epigenetic multiplayer coordinating senescence, hormone signaling, and metastasis. Cancer Sci. 2020;111(8):2718‐2725. doi:10.1111/cas.14534 32536008 PMC7419058

[18] Chen S , Ma S , Yan J , et al. Pan‐cancer analyses reveal oncogenic role and prognostic value of F‐box only protein 22. Front Oncol. 2021;11:790912. doi:10.3389/fonc.2021.790912 35141150 PMC8818750

[19] Tian X , Dai S , Sun J , et al. F‐box protein FBXO22 mediates polyubiquitination and degradation of KLF4 to promote hepatocellular carcinoma progression. Oncotarget. 2015;6(26):22767‐22775. doi:10.18632/oncotarget.4082 26087183 PMC4673198

[20] Zhang L , Chen J , Ning D , et al. FBXO22 promotes the development of hepatocellular carcinoma by regulating the ubiquitination and degradation of p21. J Exp Clin Cancer Res. 2019;38(1):101. doi:10.1186/s13046-019-1058-6 30808376 PMC6390379

[21] Zheng Y , Chen H , Zhao Y , et al. Knockdown of FBXO22 inhibits melanoma cell migration, invasion and angiogenesis via the HIF‐1alpha/VEGF pathway. Investig New Drugs. 2020;38(1):20‐28. doi:10.1007/s10637-019-00761-z 30887251

[22] Zhu XN , He P , Zhang L , et al. FBXO22 mediates polyubiquitination and inactivation of LKB1 to promote lung cancer cell growth. Cell Death Dis. 2019;10:486. doi:10.1038/s41419-019-1732-9 31217475 PMC6584689

[23] Lignitto L , LeBoeuf SE , Homer H , et al. Nrf2 activation promotes lung cancer metastasis by inhibiting the degradation of Bach1. Cell. 2019;178(2):316. doi:10.1016/j.cell.2019.06.003 31257023 PMC6625921

[24] Hou XK , Mao JS . Long noncoding RNA SNHG14 promotes osteosarcoma progression via miR‐433‐3p/FBXO22 axis. Biochem Biophys Res Commun. 2020;523(3):766‐772. doi:10.1016/j.bbrc.2020.01.016 31948764

[25] Lin M , Zhang J , Bouamar H , Wang Z , Sun LZ , Zhu X . FBXO22 promotes cervical cancer progression via targeting p57(Kip2) for ubiquitination and degradation. Cell Death Dis. 2022;13(9):805. doi:10.1038/s41419-022-05248-z 36127346 PMC9489770

[26] Qiu E , Gao Y , Zhang B , Xia T , Zhang Z , Shang G . Upregulation of cell division cycle 20 in cisplatin resistance‐induced epithelial‐mesenchymal transition in osteosarcoma cells. Am J Transl Res. 2020;12(4):1309‐1318. 32355543 PMC7191160

[27] Weng H , Xiong KP , Wang W , et al. Aspartoacylase suppresses prostate cancer progression by blocking LYN activation. Mil Med Res. 2023;10(1):25. doi:10.1186/s40779-023-00460-0 37271807 PMC10240701

[28] Wang Q , Wu L , Cao R , et al. FBXO45 promotes the malignant development of esophageal squamous cell carcinoma by targeting GGNBP2 for ubiquitination and degradation. Oncogene. 2022;41(43):4795‐4807. doi:10.1038/s41388-022-02468-7 36127399

[29] Chen T , Liu J , Zhang H , Li J , Shang G . Long intergenic noncoding RNA00265 enhances cell viability and metastasis via targeting miR‐485‐5p/USP22 axis in osteosarcoma. Front Oncol. 2022;12:907472. doi:10.3389/fonc.2022.907472 35692754 PMC9179024

[30] Ma ZQ , Feng YT , Guo K , et al. Melatonin inhibits ESCC tumor growth by mitigating the HDAC7/beta‐catenin/c‐Myc positive feedback loop and suppressing the USP10‐maintained HDAC7 protein stability. Mil Med Res. 2022;9:54. doi:10.1186/s40779-022-00412-0 36163081 PMC9513894

[31] Chen K , Wang Y , Dai X , et al. FBXO31 is upregulated by METTL3 to promote pancreatic cancer progression via regulating SIRT2 ubiquitination and degradation. Cell Death Dis. 2024;15(1):37. doi:10.1038/s41419-024-06425-y 38216561 PMC10786907

[32] Shang G , Ma X , Lv G . Cell division cycle 20 promotes cell proliferation and invasion and inhibits apoptosis in osteosarcoma cells. Cell Cycle. 2018;17(1):43‐52. doi:10.1080/15384101.2017.1387700 28980876 PMC5815435

[33] Wu L , Yu K , Chen K , et al. FBXO45 facilitates pancreatic carcinoma progression by targeting USP49 for ubiquitination and degradation. Cell Death Dis. 2022;13(3):231. doi:10.1038/s41419-022-04675-2 35279684 PMC8918322

[34] Lin X , Wang F , Chen J , et al. N(6)‐methyladenosine modification of CENPK mRNA by ZC3H13 promotes cervical cancer stemness and chemoresistance. Mil Med Res. 2022;9(1):19. doi:10.1186/s40779-022-00378-z 35418160 PMC9008995

[35] Wang L , Lin M , Chu M , et al. SPOP promotes ubiquitination and degradation of LATS1 to enhance kidney cancer progression. EBioMedicine. 2020;56:102795. doi:10.1016/j.ebiom.2020.102795 32460168 PMC7248661

[36] Li M , Zhao X , Yong H , et al. FBXO22 promotes growth and metastasis and inhibits autophagy in epithelial ovarian cancers via the MAPK/ERK pathway. Front Pharmacol. 2021;12:778698. doi:10.3389/fphar.2021.778698 34950036 PMC8688818

[37] Ge MK , Zhang N , Xia L , et al. FBXO22 degrades nuclear PTEN to promote tumorigenesis. Nat Commun. 2020;11(1):1720. doi:10.1038/s41467-020-15578-1 32249768 PMC7136256

[38] Orea‐Soufi A , Paik J , Braganca J , Donlon TA , Willcox BJ , Link W . FOXO transcription factors as therapeutic targets in human diseases. Trends Pharmacol Sci. 2022;43(12):1070‐1084. doi:10.1016/j.tips.2022.09.010 36280450 PMC12194985

[39] Chang YW , Zhao YF , Cao YL , et al. Liver X receptor alpha inhibits osteosarcoma cell proliferation through up‐regulation of FoxO1. Cell Physiol Biochem. 2013;32(1):180‐186. doi:10.1159/000350134 23867395

[40] Pei H , Jin Z , Chen S , Sun X , Yu J , Guo W . MiR‐135b promotes proliferation and invasion of osteosarcoma cells via targeting FOXO1. Mol Cell Biochem. 2015;400(1–2):245‐252. doi:10.1007/s11010-014-2281-2 25416447

[41] He W , Feng L , Xia D , Han N . MiR‐374a promotes the proliferation of human osteosarcoma by downregulating FOXO1 expression. Int J Clin Exp Med. 2015;8(3):3482‐3489. 26064239 PMC4443073

[42] Shang Y , Wang LQ , Guo QY , Shi TL . MicroRNA‐196a overexpression promotes cell proliferation and inhibits cell apoptosis through PTEN/Akt/FOXO1 pathway. Int J Clin Exp Pathol. 2015;8(3):2461‐2472. 26045752 PMC4440061

[43] Guan H , Tan P , Xie L , et al. FOXO1 inhibits osteosarcoma oncogenesis via Wnt/beta‐catenin pathway suppression. Oncogene. 2015;4(9):e166. doi:10.1038/oncsis.2015.25 PMC4767937 26344693

[44] Li S , He J , Liao X , et al. FBXO22 inhibits metastasis in triple‐negative breast cancer through ubiquitin modification of KDM5A and regulation of H3K4me3 demethylation. Cell Biol Toxicol. 2022;39:1641‐1655. doi:10.1007/s10565-022-09754-w 36112263 PMC10425479

[45] Guo F , Liu J , Han X , et al. FBXO22 suppresses metastasis in human renal cell carcinoma via inhibiting MMP‐9‐mediated migration and invasion and VEGF‐mediated angiogenesis. Int J Biol Sci. 2019;15(3):647‐656. doi:10.7150/ijbs.31293 30745851 PMC6367582

[46] Wu B , Liu ZY , Cui J , et al. F‐box protein FBXO22 mediates polyubiquitination and degradation of CD147 to reverse cisplatin resistance of tumor cells. Int J Mol Sci. 2017;18(1):212. doi:10.3390/ijms18010212 28117675 PMC5297841

[47] Nie DY , Tabor JR , Li J , et al. Recruitment of FBXO22 for targeted degradation of NSD2. Nat Chem Biol. 2024. doi:10.1038/s41589-024-01660-y PMC11581931 38965384

[48] Basu AA , Zhang C , Riha IA , et al. A CRISPR activation screen identifies FBXO22 supporting targeted protein degradation. Nat Chem Biol. 2024. doi:10.1038/s41589-024-01655-9 PMC11581908 38965383

[49] Kagiou C , Cisneros JA , Farnung J , et al. Alkylamine‐tethered molecules recruit FBXO22 for targeted protein degradation. Nat Commun. 2024;15(1):5409. doi:10.1038/s41467-024-49739-3 38926334 PMC11208438

[50] Sun R , Xie HY , Qian JX , et al. FBXO22 possesses both protumorigenic and antimetastatic roles in breast cancer progression. Cancer Res. 2018;78(18):5274‐5286. doi:10.1158/0008-5472.CAN-17-3647 29945959

[51] Johmura Y , Maeda I , Suzuki N , et al. FBXO22‐mediated KDFM4B degradation determines selective estrogen receptor modulator activity in breast cancer. J Clin Invest. 2018;128(12):5603‐5619. doi:10.1172/JCI121679 30418174 PMC6264734

[52] Bai J , Wu K , Cao MH , et al. SCF(FBXO22) targets HDM2 for degradation and modulates breast cancer cell invasion and metastasis. Proc Natl Acad Sci USA. 2019;116(24):11754‐11763. doi:10.1073/pnas.1820990116 31138683 PMC6575577

[53] De S , Holvey‐Bates EG , Mahen K , Willard B , Stark GR . The ubiquitin E3 ligase FBXO22 degrades PD‐L1 and sensitizes cancer cells to DNA damage. Proc Natl Acad Sci USA. 2021;118(47):e2112674118. doi:10.1073/pnas.2112674118 34795058 PMC8617495

[54] Staron MM , Gray SM , Marshall HD , et al. The transcription factor FoxO1 sustains expression of the inhibitory receptor PD‐1 and survival of antiviral CD8^+^ T cells during chronic infection. Immunity. 2014;41(5):802‐814. doi:10.1016/j.immuni.2014.10.013 25464856 PMC4270830

[55] Lu Y , Zhang J , Chen Y , et al. Novel immunotherapies for osteosarcoma. Front Oncol. 2022;12:830546. doi:10.3389/fonc.2022.830546 35433427 PMC9012135

[56] Vrba L , Junk DJ , Novak P , Futscher BW . p53 induces distinct epigenetic states at its direct target promoters. BMC Genomics. 2008;9:486. doi:10.1186/1471-2164-9-486 18922183 PMC2585595

[57] He Y , Wang Y , Liu L , et al. Circular RNA circ_0006282 contributes to the progression of gastric cancer by sponging miR‐155 to upregulate the expression of FBXO22. Onco Targets Ther. 2020;13:1001‐1010. doi:10.2147/OTT.S228216 32099403 PMC6999548

[58] Liu J , Shang G . The roles of noncoding RNAs in the development of osteosarcoma stem cells and potential therapeutic targets. Front Cell Dev Biol. 2022;10:773038. doi:10.3389/fcell.2022.773038 35252166 PMC8888953

